# Relationships between Contextual and Task Performance and Interrater Agreement: Are There Any?

**DOI:** 10.1371/journal.pone.0139898

**Published:** 2015-10-16

**Authors:** Luis F. Díaz-Vilela, Naira Delgado Rodríguez, Rosa Isla-Díaz, Dolores Díaz-Cabrera, Estefanía Hernández-Fernaud, Christian Rosales-Sánchez

**Affiliations:** Department of Cognitive, Social and Organizational Psychology, La Laguna University, San Cristóbal de La Laguna, Spain; University of Reading, UNITED KINGDOM

## Abstract

Work performance is one of the most important dependent variables in Work and Organizational Psychology. The main objective of this paper was to explore the relationships between citizenship performance and task performance measures obtained from different appraisers and their consistency through a seldom-used methodology, intraclass correlation coefficients. Participants were 135 public employees, the total staff in a local government department. Jobs were clustered into job families through a work analysis based on standard questionnaires. A task description technique was used to develop a performance appraisal questionnaire for each job family, with three versions: self-, supervisor-, and peer-evaluation, in addition to a measure of citizenship performance. Only when the self-appraisal bias is controlled, significant correlations appeared between task performance rates. However, intraclass correlations analyses show that only self- (contextual and task) performance measures are consistent, while interrater agreement disappears. These results provide some interesting clues about the procedure of appraisal instrument development, the role of appraisers, and the importance of choosing adequate consistency analysis methods.

## Introduction

The main objective of this paper was to explore the relationships between citizenship performance and task performance measures obtained from different appraisers and the consistency of scores between raters through a seldom-used methodology, intraclass correlation coefficients.

Work performance is one of the most important dependent variables in Work and Organizational Psychology [1, 2]. It is increasingly important to expand the scope of performance appraisal to all behaviors that have an impact on organizational outcomes, including task-specific and discretionary work behaviors [3, 4, 5].

One general definition of work performance includes those quantifiable employee behaviors and outcomes that contribute to organizational goals [6]. Smith [7] established an important distinction between behaviors, outcomes, and organizational effectiveness, the first two being the cause of the latter. From Campbell’s perspective [8, 9, 10, 11], individual performance is defined as particular behaviors that can be observed and measured in terms of skills and abilities, with less emphasis on organizational outcomes. Thus, work performance includes those behaviors that are relevant to organizational goals, are under individual control, and can be observed and measured.

During the last two decades or so, an important distinction has been established between two types of work performance: task and contextual performance. The former refers to the prescribed role an employee should comply with in order to attain organizational goals. It can be defined as the efficacy with which incumbents perform activities that contribute to the development of the organization’s technical core. This contribution can be direct, including the application of a part of organizational technology, or indirect, providing materials or services needed to perform organizational technical processes [12]. Contextual performance, also called citizenship performance, involves those behaviors not directly related to job tasks, but having a significant impact on organizational, social, and psychological contexts. These behaviors serve as catalyzers for the efficient undertaking of the entrusted tasks. Borman and Motowidlo [13] proposed a model that included five types of citizenship behaviors: persisting with enthusiasm and extra effort to complete one’s task activities; volunteering to carry out task activities that are not part of one’s job; helping and cooperating with others; following organizational rules and procedures; and endorsing, supporting, and defending organizational objectives. In this kind of performance the initiative, support, and persistence that employees demonstrate is more important than the technical competence displayed [14]. Contextual or civic activities support and create the context or social environment in which the technical core of the organization must function, while task activities serve to support and create the technical core itself [15].

Given that task and contextual performance are two major dimensions of individual work performance, and attending to their nature and their contributive role in organizational goals attainment, a certain relationship between them is expected. In a recent meta-analysis, whose objective was to shed light on the relationship of contextual performance with individual and organizational consequences, a moderate correlation of .40 was found between task and contextual performance [16]. Podsakoff et al. [16] examined the potential impact of same-source biases on the relationships between task and overall job performance ratings and between contextual and overall job performance. Their results showed that the overall relationship between Organizational Citizenship Behavior (OCB) and job performance was significantly stronger when measures were taken from the same source (r = .62) than when they were obtained from different sources (r = .32). OCB and job performance ratings shared about three to four times more variance when they came from the same source (36%–48%) than when they were obtained from different sources (8%–13%). These authors did not explore how the relationship between task and contextual performance is affected by the rating source. Nevertheless, this relationship can be expected to decline when raters are different individuals. Hence our first hypotheses are:

Hypothesis 1a: OCB and task performance will correlate significantly, and

Hypothesis 1b: This relationship will be stronger when both measures come from the same source.

As Murphy and De Shon [17] state, severe range restriction and leniency can reduce the size of interrater correlations in performance measures. If this statement is true, interrater correlations should be higher when raters are not concentrating their ratings at the top-end of the scale: lower range restriction and less leniency (raters with lower means and larger standard deviations). Hence our second hypothesis:

Hypothesis 2: Less lenient and less range restricted cases will show higher interrater consistency.

In relation to performance measurement, Viswesvaran and Ones [6] provide a classification framework according to two dimensions. The first has to do with the developmental context of job performance appraisal and has two categories: stand-alone specific, or part of a larger set of dimensions. The second classifying dimension refers to the span of application and has two levels: limited to specific occupations or job families, or applicable across jobs.

Appraising models centered on job families or specific occupations, and based upon stand-alone measures, are grounded in work analysis and description. Measures are developed from task inventories or job specifications. These techniques result in ad hoc performance profiles, which are particular to the situation under investigation. They are appropriate when there is substantial knowledge of the transformation process of tasks under evaluation. Although several authors point out the importance of considering the nature of the task, matching this nature with the performance appraisal format [18, 19], research reporting a work analysis is hard to come by. Moreover, as Hoffman, Blair, Meriac, and Woehr [3] report, none of their 112 revised studies conceptualized task (and citizenship) performance multidimensionally. Regarding this topic, this work provides a comprehensive procedure to develop job family-specific measures of job performance.

The opposite type of appraising models brings together those interested in sets of dimensions applied across occupations [9, 6, 20]. Within this approach, Borman, Penner, Allen, and Motowidlo [21] give a revised taxonomy of three general dimensions that compose the organizational citizenship performance domain. These dimensions are personal support, organizational support, and conscientious initiative. Empirical research on this model resulted in two types of conclusions. Some researchers found a single factor [3, 22], while others found two stable dimensions representing Borman et al.’s [21] personal and organizational support. Conscientious initiative has seldom received empirical support [23, 24, 25]. Moreover, some research gives evidence of the existence of only two citizenship dimensions: organization-related and peer-related [26, 27, 28]. Podsakoff, Whiting, Podsakoff, and Blume [16] point out that this two dimensional model offers a good conceptualization of the citizenship performance domain. Regarding this topic, this study addresses contextual performance measures from a dimensional approach.

The very nature of both types of work performance (task and contextual) compels to develop domain-specific appraisal procedures. In general, contextual or civic performance does not need job-specific instruments or methods, but the same instrument can and should be used across jobs. On the contrary, task performance appraisal may require focusing the procedure on formal aspects of each job: different questionnaires for different jobs. Task performance assessment within this category requires a previous job analysis and description, and a decision on which criteria should be included for each job. This approach would produce as many appraisal criteria (appraising questionnaires) as existing jobs, making the process very complex and expensive. One solution to this problem can be clustering jobs into job families to produce fewer job family-specific questionnaires, and adopting a model with an occupational focus limited to these job families [6]. The problem here is the standardization of performance scores across jobs or job families. That is, because measures come from different questionnaires, equalities of means and variances across measures must be proven. As task performance measure was job family-specific oriented in this study, equality of means and homogeneity of variances will be tested:

Hypothesis 3: Task performance means and variances will be equal across job families. The use of job-family specific measures constrains the testing of inter-family equalization of results: namely, analyzing convergence of data distributions across job families.

Another related question is bound up with sources of evaluation information. Differential validity can be assessed by estimating correlations among different sources, or by analyzing correlation patterns between scores obtained through different sources with other external variables [2]. The former option focuses on internal construct consistency when different sources evaluate it, while the latter explores the cross-structure of performance measures as compared with other constructs [29]. Although there are a number of theoretical mechanisms proposed to explain why supervisor, peer, and self-appraisals could differ (for instance, different appraisers have different goals or opportunities to observe incumbent’s behavior), empirical evidence shows that discrepancy between sources is not especially relevant, with moderate correlations between .52 and .74 [2]. Traditionally, interrater correlation was interpreted as agreement, while deviations from perfect correlation was interpreted as measurement error. A meta-analysis carried out by Viswesvaran, Schmidt, and Ones [30] on observed correlations between supervisor and peer ratings for different dimensions of job performance, including task performance, found that the overlap between raters was substantial. However, other indices seem to be more suitable for testing whether all appraisers are assessing the same dimensions with the same value appreciation: Intraclass correlation coefficient [31], or ICC; concordance correlation coefficient [32], nearly identical to ICC; Cohen’s kappa statistic [33], for categorical items and two raters; or Fleiss’s kappa [34] for categorical items and several raters. Unfortunately, little or no research in this field reports results based upon consistency coefficients within the framework of ANOVA or random effects models [35]. This issue is addressed in the current study by providing a different perspective on the analysis of appraisers’ consistency, using intraclass correlation coefficients [31]. One special case of these coefficients is ICC(3), which should be applied when a fixed set of k judges rate each target and raters are seen as random effects. In this case, mean differences between judges are removed, and therefore ICC(3) is sensitive to variance differences between judges [36].

## Method

### Participants

Participants of this study were 135 public employees (29.6% males and 70.4% females), working in a local government department. The department’s main function is to develop and implement studies and proposals for the autonomous or regional government in matters related to tourism, as well as coastal planning, promotion, and infrastructure. These 135 employees represent the total number of staff in the department, which is structured into 18 units (services, general directorates, and supporting units), and 25 different jobs. These jobs were clustered into eight families: Administrative clerks (37%), Riggers or Construction Engineers (2.2%), Inspectors (3.7%), Section/Service Heads (22.2%), Computer Operators (3.7%), Secretaries (4.4%), Subordinates (8.9%), and Law graduates (17.8%).

Supervisors and peers, who participated in a voluntary manner, were asked to appraise these 135 employees. Unfortunately, only seven (38.89%) supervisors appraised 66 (48.89%) subordinates, while 22 peers from nine services appraised one of their colleagues (16.30%)

### Instruments

Two instruments were used in this research. One was developed ad hoc to evaluate task performance and had three versions: self-, supervisor-, and peer-evaluator. The second was a Spanish adaptation of Coleman and Borman’s [23] Organizational Citizenship Behavior (OCB) questionnaire [24].

#### Task performance questionnaire

Task performance questionnaires used in this study were ad hoc instruments that resulted from a work analysis process based upon structured (questionnaires) and semi-structured techniques (guided interviews). Structured questionnaires were used to form job families prior to the development of as many task-based appraisal questionnaires as families were found. This developing process consisted of three phases:

Job Analysis Phase: The job analysis phase consisted of developing a job description, applying an adaptation into Spanish of O*NET instruments [37] to the sample, and presenting a Spanish version of the Position Analysis Questionnaire (PAQ) [38] to expert analysts.

To generate task-based job descriptions, employees answered an open questionnaire in which they were asked to list their most important functions and about eight tasks related to each function. Once these data were analyzed, a job analysis interview was held with each incumbent to clarify doubts and incongruences between standard (O*NET) and open questionnaires.

Parallel to this process, each job was analyzed by two technicians who answered the PAQ questionnaire separately and were then required to reach consensus on their scores. A cluster analysis was performed on PAQ second order dimensions (dimensions 33 to 45) to form preliminary groups. After an expert panel discussion using O*NET, job descriptions, and PAQ results, jobs were finally clustered into the eight families listed above.

Task Inventory Phase: Task inventories were family-specific and were developed from job descriptions. In a first step, task inventories included all tasks from all jobs within each family. Task inventories were administered to all the incumbents, who scored each task according to frequency, importance, and complexity criteria. All tasks applicable to at least one job within the family (at least one employee declared having performed it) were selected to devise the appraisal questionnaire. Each family had a specific inventory and thus a different questionnaire: a different number of tasks and different tasks.

Phase of development of the task performance questionnaire: Once task inventories were developed, a subject matter expert (SME) board elaborated the task performance appraisal questionnaires. This SME board included researchers and administration experts, supervisors, and union representatives, who revised each inventory and eliminated or included some tasks following a relevance criterion. Finally jobs within each family shared the following number of tasks: Administrative clerks (60); Riggers or Construction Engineers (17), Inspectors (26), Section/Service Heads (77), Computer Operators (24), Secretaries (34), Subordinates (31), and Law graduates (54).

Job family-specific questionnaires included items formulated from each task specification. The descriptive statement for each task was formulated with an evaluative behavioral assertion aimed at discovering workers’ task performance. For instance, the task “Updates records with new documents” produced the performance assertion “Updates records with new documents regularly and correctly”. Each of these assertions had a graphic ten-point scale with five anchorages, from “Almost never” to “Always”, plus a “Does not apply” box. For each case, the task performance score was computed by averaging answers to the worker’s particular number of responses.

#### Contextual performance questionnaire

Contextual performance was assessed through a Spanish adaptation of Coleman and Borman’s [23] questionnaire. This version had 27 items with six-point graphic scales and two extreme anchors that can be summarized in a general single dimension [24]. According to these authors’ results, this questionnaire addresses behaviors aimed at benefiting other individuals and the organization, although a single-factor solution is sufficiently reliable. Participants were asked to answer each question depending on what they believed better described their behavior at work. In order to make Task and Contextual performance scores comparable in range, and means, OCB item responses were multiplied by their factor coefficients from a principal components analysis (forced to a single component solution). These scores were summed up and transformed so that the minimum possible (27 raw points) became 0 and the maximum (27*6 raw points) became 10, following the formula:$\left[\left(\sum_{i=1}^{27}x_{i}\times Fc_{i}\right)-1.44\right]\;*.44ipli$. Where *x*_*i*_ is the raw answer to item *i*, and *Fc*_*i*_ is the factor coefficient of that item. Correlation between raw score means and this OCB score was r_xy_ = 1.

### Procedure

Each employee received a set of questionnaires that included his or her job family task performance questionnaire, and a contextual performance questionnaire. After around two weeks, a researcher collected the questionnaires in a sealed envelope in order to guarantee confidentiality.

On a second wave of data collection, each employee was assigned a peer to evaluate through the same task performance appraisal. Peer appraisers were randomly assigned whenever possible, given that each appraiser would not be evaluated back by his/her appraisee. Supervisors corrected assignments when social problems were expected. Therefore, for each unit, all the employees were assigned an appraisee within the unit. Once again, employees had around two weeks to complete the questionnaire. Unfortunately, due to the reticence of union representatives, neither supervisors nor peers evaluated workers’ OCB.

Alongside these data collections, supervisors answered a third version of the family-specific task performance questionnaire. Each supervisor answered as many questionnaires as employees in his or her unit. These questionnaires were the same as those used for the self-appraisals but items were expressed in the third person.

In summary, each employee answered a (job family-specific) task performance and a contextual performance self-evaluation questionnaire, plus a (peer’s job family-specific) task performance peer-evaluation questionnaire. Supervisors answered as many supervisor-evaluation (employees’ job families-specific) questionnaires as employees were in their units.

Participants were informed individually and confidentially about their task and contextual scores. When both supervisor and peer rated a participant, the average score of both raters was also included. Participants were also informed about the average scores of their work unit and organization. Finally, overall results were reported to the organization maintaining confidentiality of individual participants. This feedback procedure was negotiated previously with workers representatives and the decision was taken by the SME board.

### Ethics Statement

Because the study involved no risk to participants, informed consent was given verbally. Participants were clearly informed that the participation was voluntary and that there would be no compensation for participation. The University of La Laguna Ethics Committee in Tenerife, Spain (ULLECT) approved this study. Besides, administration experts, supervisors, and union representatives, as members of the evaluation committee, supervised voluntariness of participants. Thus, consent was implicit when a worker, co-worker, or supervisor participated. The ULLECT approved this procedure a posteriori, while Administration managers, Unions representatives, and workers themselves approved it verbally in previous several sectorial and general meetings. Finally, the University of La Laguna Ethics Committee approved the consent procedures.

## Results

Table 1 shows the distribution statistics of the four dependent variables. All the distributions were right-handed asymmetric except for task performance peer evaluations. Means are all high, more than two points over the central point of the scale (5.0). However, tests of normality of distributions (diagonal in the matrix) were not significant.

**Table 1:** Distribution statistics, correlations, and F tests across measures of the four dependent variables.

							Asymmetry		Kurtosis		Correlationsa / Normality / F tests				
		N	Min	Max	Mean	S.D.		S.E.		S.E.		1	2	3	4
1	OCB	135	2.38	10.00	7.29	1.45	-0.366	0.209	-0.126	0.414	0.698	0.698	1.275	4.227***	0.063
2	Task self	135	3.64	10.00	7.16	1.36	-0.101	0.209	-0.375	0.414	0.563	0.563***	0.528	12.586**	0.031
3	Task supervisor	66	4.82	10.00	7.86	1.64	-0.292	0.295	-1.201	0.582	0.116	0.116	0.193	0.819	3.321
4	Task peer	22	4.83	9.88	7.31	1.34	0.255	0.491	-0.494	0.953	0.106	0.106	0.137	0.058	0.528

* p ≤ .05,

** p ≤ .01,

*** p ≤ .001. a D.f. for each correlation is the lowest N of variables involved. Correlations in the lower triangle Kolmogorov-Smirnov Z tests in the diagonal; F tests of equality of means in the upper triangle.

As Table 1 shows, task and contextual performance self-evaluations correlate significantly (r_xy_ = .563; p ≤ .001), as formulated in Hypothesis 1. However, no significant correlations were found between self-evaluations and others’ evaluations. Intraclass correlation coefficients shown in table 2 replicate Pearson correlations. Only self-evaluations correlate significantly (ICC = .719; *p* ≤ .001). These results contrast with previous research, and support the first hypothesis only when OCB and task performance are self-evaluated. They also support our Hypothesis 1b in that relationships are stronger when ratings come from the same source measures than when they come from different sources. Moreover, these relationships disappear when sources are different individuals.

**Table 2:** Intraclass correlation coefficients between performance measures.

	d.f.	OCB	Task self	Task supervisor
Task self	135	.719***		
Task supervisor	66	.168	.193	
Task peer	22	.221	.275	-.018^a^

***p ≤ .001;

^a^d.f. = 17. Overall consistency for three raters in task performance measures: ICC (3) = .303; n.s.; N = 17. ICC’s were calculated with “irr” package within R, assuming a one-way model (row effects random), and an average unit of analysis.

Given that all participants answered the same OCB questionnaire (Task performance questionnaires were job family-specific), subjects were grouped depending on their response tendency in this questionnaire: those tending to score on top with low standard deviations *vs* those without this tendency and larger standard deviations. For each case, mean and standard deviation of his/her answers to the 27 items in the questionnaire were computed. Next, an iterative K means cluster analysis grouped cases with high right-handed tendency in one cluster (Centered around an average mean of $\bar{x}$ = 8.46, and an average standard deviation of SD = .76; n = 69), and those without this tendency in another (Centered around an average mean of $\bar{x}$ = 6.07, and an average standard deviation of SD = .85; n = 66).

The lower triangles of the matrices displayed in Table 3 show correlation results among performance variables within each of these clusters. In the not-so-high OCB group (Cluster 2), correlations are positive and significant, except for the pair supervisors-peers, where no significance was reached and the sample size was too small. These results with the not-so-high OCB cluster are in line with previous research and support our first and second hypotheses, since we expected significant correlations between every pair of performance variables. In the high OCB group these correlations vanished, resulting in only a small but significant correlation between self-OCB and task performance evaluations (r_xy_ = .263; *p* ≤ .05).

**Table 3:** Correlations and intraclass consistency coefficients between performance measures within each OCB Cluster.

	OCB	Task—self	Task—supervisor	Task—peer
Cluster 1 –High OCB ($\bar{x}$ = 8.46)				
OCB		.191	-.062	-2.680
Task self (69)	.263*		.201	.070
Task supervisor (35)	.025	.113		-1.890^a^
Task peer (11)	.062	.200	-.661^a^	
Cluster 2 –Not-so-high OCB ($\bar{x}$ = 6.07)				
OCB		.572***	-.122	.379
Task self (66)	.474***		.092	.351
Task supervisor (31)	.451**	.448**		.671^b^
Task peer (11)	.817**	.594*	.547^b^	

*p ≤ .05,

**p ≤ .01,

***p ≤ .001. Degrees of freedom in parentheses.

^a^ n = 9;

^b^ n = 8. Pearson correlations in the lower triangle and intraclass correlations in the upper triangle of each matrix. High OCB cluster’s overall task performance consistency: ICC(3) = -.260; n.s.; N = 9. Not-so-high OCB cluster’s overall task performance consistency: ICC(3) = .614; p ≤ .05; N = 8. ICC’s were calculated with “irr” package within R, assuming a one-way model (row effects random), and an average unit of analysis.

However, results with intraclass correlation coefficients are quite different. Upper triangles of the matrices displayed in Table 3 show that consistency appears only when measures come from workers themselves (OCB *vs* task self-), and, in this case, only in the not-so-high OCB group (ICC(2) = .572; *p* ≤ .001). For the remaining measures no consistency was found, not even in the case of OCB and self-task within the high OCB group. These results do not support the first and second hypotheses except for the case of self-reported evaluations in the not-so-high OCB group.

Mean differences across measures were significant when considering supervisors’ evaluations. Table 1 showed that supervisors’ rates were higher than employees’ self-appraisals (Task *vs*. OCB: F (1, 65) = 4.227; *p* ≤ .05; η^2^ = .061. Task *vs*. Task: F (1, 65) = 12.586; *p* ≤ .01; η^2^ = .162), and peer appraisals, although in this case the difference was not significant (F (1, 16) = 3.321; n.s.; η^2^ = .172). Peer and self-appraisals resulted in equal means (F (1, 21) = .031; n.s.; η^2^ = .001).

Levene’s tests in Table 4 show that variances are homogeneous across families for all variables except for supervisors’ appraisals (F (5, 65) = 4.286; *p* ≤ .01). Likewise means are equal across job families, except when supervisors are the raters (F (5, 65) = 6.136; *p* ≤ .001; η^2^ = .338). Aside from the generally higher supervisors’ mean and the non-homogeneity of variances and mean differences in supervisors’ evaluations across families, the questionnaires appear to be working well. However, supervisors responded in a non-standard way. Our third hypothesis was partially confirmed, but supervisors’ behavior warranted more analysis.

**Table 4:** ANOVA tests of between job-families equality of means and Levene’s tests of homogeneity of variances.

	d.f.	F	η^2^	Obs. Power^1^	Levene’s test (d.f.)	Levene’s test (F)	Adj. R^2^
OCB	7, 134	1.484	.076	.606	7,127	1.651	.034
Task Self	7, 134	1.856	.093	.723	7,127	.660	.051
Task Supervisor	5, 65	6.136***	.338	.993	5,65	4.286**	.629
Task peer	6, 21	.980	.282	.272	6,18	.754	.000

Independent variable: Job family.

** p ≤ .01;

*** p ≤ .001.

^1^ Power was computed for α = .05.

Such significance disappeared when we controlled for job family effects on supervisor effects. An ANOVA applied to supervisors’ evaluations, depending on supervisors themselves and on job families, indicates that the real effect comes from supervisors (different assessors) (F (7, 65) = 7.334; *p* ≤ .001; η^2^ = .517), not from job families (different questionnaires) (F (5, 65) = .424; *n*.*s*.; η^2^ = .042), and that these variables do not interact (F (5, 65) = .913; *n*.*s*.; η^2^ = .087).

This effect can therefore be associated with the idiosyncrasies of supervisors when answering the questionnaire. Non-homogeneity of variances reduces the significance of these results, but the effect size points to a real effect of supervisor.

## Discussion

This study focuses on the analysis of the relationships between task and citizenship performance and between raters, using Pearson and intraclass correlation coefficients. The main objective was to explore the relationship of citizenship performance with task performance measures obtained from different raters and the consistency of scores between raters. Results provide some interesting clues about the role of measurement instruments and pinpoints certain problems related to extremely high answers. Specifically, there is a relationship between task and citizenship performance self-appraisals. Nevertheless relationships between self- and peer/supervisors’ performance evaluations seem to be mediated by other factors. Namely, interrater correlations exist when the appraisee does not show a tendency to a very high self-evaluation. On the other hand, intraclass correlations show that, although this relationship can exist there is no interrater consistency.

The first hypothesis stated that OCB and task performance would correlate significantly. Results partially support this hypothesis, in line with Podsakoff et al.’s [16] meta-analysis findings. Thus, a high positive correlation appeared between task and contextual performance when the evaluations came from the appraisee. However, interrater correlations did not support this hypothesis when the total sample was analyzed, in contrast to previous findings [2, 30]. Results from Pearson and intraclass correlation analyses are very similar. Deeper scrutiny of this sample showed that those who made more reasonable (not-so-high) OCB performance self-evaluations had higher correlations (OCB—task self-reported, and OCB—task others rates). However, intraclass consistency appeared only for self-evaluations, especially in the not-so-high OCB group. These results provide evidence to confirm our second hypothesis (less lenient and less range restricted cases will show higher interrater correlations) and show the importance of controlling contamination due to social desirability and/or leniency in performance measures. This concurs with previous research finding of correlation between raters expressing a combination of factors with different theoretical relationships with validity [39, 17]. In this case, self-leniency may be one of these factors. Intraclass analyses strengthen this interpretation. Although correlations may be significant, consistency is not present because of differences in response behavior. Different raters are therefore rating against different standards. To illustrate this, we consider three hypothetical cases where 135 workers self-report their performance (*y)* and obtain rates from their supervisors (*x)*. When raters differences are systematically of one point (*y* = *x* + 1), of two points (*y* = *x* + 2), or double (*y* = *x* × 2), classic interrater reliability would be perfect in all cases, r_xy_ = 1. But consistency undergoes severe variations (ICC = .881; *p* ≤ .001, ICC = .522; *p* ≤ .001, and ICC = -1.93; *p* = 1, respectively).

On the other hand, an analysis of distributions shows that graphic Behavioral Observation Scales (BOS) are by nature right-biased. Tasks presented in the appraising instrument seem to be pertinent, since they result from job analysis. Questions format, however, should make it more difficult to give a biased answer.

The third hypothesis stated that means and variances would be homogeneous across job families, even though questionnaires were job family-specific and participants answered them from a job perspective (only answering those questions that applied to their jobs). In general terms, results support this assertion even in the case of supervisors’ evaluations, where supervisor style effect made job family effect negligible. From this finding, we can conclude that the effects do not arise from the questionnaire itself, but from supervisors’ answering styles. These effects could be reduced by training supervisors as appraisers, by penalizing lenient supervisors [40, 41], or, perhaps, by reinforcing those good appraisers.

Overall, the results of this study constitute an important contribution because they demonstrate that self-leniency modifies the relationships between different raters’ appraisals. On the other hand, our results advise against the use of Pearson correlations as interrater reliability estimators and encourages future researchers to use appropriate statistical techniques. The title of this paper questions whether any of the predicted relationships exists. Given these results, the answer should be negative for different raters, unless appropriately conducted future research reveals the opposite to be true. Another important contribution of this research was the procedure used to develop task performance measures. A complex task-based procedure for detecting specific performance dimensions is unusual. Moreover, procedures oriented to clustering jobs in job families are very scarce. When job analyses are carried out correctly, an increase in the validity of questionnaires is expected.

From an applied perspective, our findings highlight the importance of standardizing responses among raters. Cardy and Dobbins [42] emphasize the relevance of reducing appraisers’ biases and enhancing measurement precision. Accordingly, whether the informants are incumbents, supervisors, or peers, training is required before measuring performance. In addition, the use of job-family specific questionnaires in performance appraisal considerably reduces time and effort without losing validity, while response scales are important sources of discrimination errors. Graphic scales do not seem to be appropriate for this, indicating that forced choice scales, or perhaps quasi-ipsative measures [43], should be the goal.

Despite several strengths, this study has its limitations. First, sample size was small (very small in some job families), and supervisor and peer participation was too low. Further work is therefore required to confirm these results. Similarly, the sample of supervisor and peer appraisers should also be larger. Since the study took place in a single organization, future research in different organizations and work settings is needed to replicate our findings, and examining the effect of supervisor style within different job contexts would be of interest. Moreover, differences between public and private organizations may influence both the size of evaluation scores and the behaviors of interest. Our finding that supervisors gave higher scores than peers and employees themselves is noteworthy. This may not be the case in private firms and may be due to non-professional leadership in the Spanish administration: however, there is no research addressing this topic. Finally, Jawahar and Ferris’ [44] results should be considered with regard to how supervisors’ appraisals of workers’ task and contextual performance covariate, and future research should also explore this relationship.
